# Supplementary material for: HIVprotI: an integrated web based platform for prediction and design of HIV proteins inhibitors
Source: J Cheminform. 2018 Mar 9;10:12. doi: 10.1186/s13321-018-0266-y (PMC5845081; doi:10.1186/s13321-018-0266-y)
Supplement: Supplementary file 2 — Additional file 2. Source code of HIVProtI web server. [file 13321_2018_266_MOESM2_ESM.zip › HIVprotI_Source-code/hivprotI/jsme/api_javadoc/export/client/Utils.html]

Utils


JavaScript is disabled on your browser.


Skip navigation links


- Package
- Class
- Tree
- Deprecated
- Index
- Help

- Prev Class
- Next Class

- Frames
- No Frames

- All Classes

- Summary:
- Nested |
- Field |
- Constr |
- Method

- Detail:
- Field |
- Constr |
- Method


export.client

## Class Utils

- java.lang.Object
- - export.client.Utils

- ---

    

  ```
  public class Utils
  extends java.lang.Object
  ```

- - ### Constructor Summary

    Constructors

    Constructor and Description

    `Utils()`
  - ### Method Summary

    All Methods Static Methods Concrete Methods

    | Modifier and Type | Method and Description |
    | --- | --- |
    | `static void` | `runAfterBrowserEventLoopReturns(JavaScriptObject jsFunction)` Run some Javascript code after the browser event loop returns. |
    | `static void` | `runAfterFixedDelay(JavaScriptObject jsFunction, int delayMS)` Run some Javascript code after the browser event loop returns. |
    | `static void` | `safeLog(java.lang.String msg)` console.log tested on Chrome with success On IE: the console is not available unless debugger is on: causes an exception. |

    - ### Methods inherited from class java.lang.Object

      `equals, getClass, hashCode, notify, notifyAll, toString, wait, wait, wait`

- - ### Constructor Detail


    - #### Utils

      ```
      public Utils()
      ```
  - ### Method Detail


    - #### runAfterBrowserEventLoopReturns

      ```
      public static void runAfterBrowserEventLoopReturns(JavaScriptObject jsFunction)
      ```

      Run some Javascript code after the browser event loop returns. Example:
      JSApplet.Utils.runAfterBrowserEventLoopReturns(myFunction);

      Parameters:
      :   `jsFunction` - : a function defined in JavaScript such as :
          function(){alert("DOM update finished")};


    - #### runAfterFixedDelay

      ```
      public static void runAfterFixedDelay(JavaScriptObject jsFunction,
                                            int delayMS)
      ```

      Run some Javascript code after the browser event loop returns. Example:
      JSApplet.Utils.runAfterBrowserEventLoopReturns(myFunction);

      Parameters:
      :   `jsFunction` - : a function defined in JavaScript such as :
          function(){alert("DOM update finished")};


    - #### safeLog

      ```
      public static void safeLog(java.lang.String msg)
      ```

      console.log tested on Chrome with success On IE: the console is not
      available unless debugger is on: causes an exception. This method
      catches the exception and ignores it.

      Parameters:
      :   `msg` -


Skip navigation links


- Package
- Class
- Tree
- Deprecated
- Index
- Help

- Prev Class
- Next Class

- Frames
- No Frames

- All Classes

- Summary:
- Nested |
- Field |
- Constr |
- Method

- Detail:
- Field |
- Constr |
- Method
